# Evaluation of Aerosol Drug Delivery Options during Adult Mechanical Ventilation in the COVID-19 Era

**DOI:** 10.3390/pharmaceutics13101574

**Published:** 2021-09-28

**Authors:** Piers J. Naughton, Mary Joyce, Marc Mac Giolla Eain, Andrew O’Sullivan, Ronan MacLoughlin

**Affiliations:** 1 School of Chemistry, National University of Ireland, H91 CF50 Galway, Ireland; p.naughton2@outlook.com; 2 Research and Development, Science and Emerging Technologies, Aerogen Limited, Galway Business Park, H91 HE94 Galway, Ireland; mjoyce@aerogen.com (M.J.); mmacgiollaeain@aerogen.com (M.M.G.E.); aosullivan@aerogen.com (A.O.); 3 School of Pharmacy & Biomolecular Sciences, Royal College of Surgeons in Ireland, D02 YN77 Dublin, Ireland; 4 School of Pharmacy and Pharmaceutical Sciences, Trinity College, D02 PN40 Dublin, Ireland

**Keywords:** aerosol, vibrating mesh nebuliser, jet nebuliser, pressurised metered-dose inhaler, spacer, adapter, mechanical ventilation, lung-protective ventilation, actuation, COVID-19

## Abstract

Drug delivery devices used for aerosol therapy during mechanical ventilation to ease the symptoms of respiratory diseases provide beneficial treatment but can also pose challenges. Reflecting the significant changes in global guidance around aerosol usage and lung-protective ventilation strategies, seen in response to the COVID-19 pandemic, for the first time, we describe the drug delivery performance of commonly used devices under these conditions. Here, vibrating mesh nebuliser (VMN), jet nebuliser (JN) and pressurised metered-dose inhaler (pMDI) performance was assessed during simulated adult mechanical ventilation. Both standard test breathing patterns and those representatives of low tidal volume (LTV) ventilation with concurrent active and passive humidification were investigated. Drug delivery using a VMN was significantly greater than that with a JN and pMDI for both standard and LTV ventilation. Humidification type did not affect the delivered dose across all device types for standard ventilation. Significant variability in the pMDI dosing was evident, depending on the timing of actuation and the adapter type used. pMDI actuation synchronised with inspiration resulted in a higher delivered drug dose. The type of adapter used for pMDI actuation influenced drug delivery, with the highest dose observed using the CombiHaler.

## 1. Introduction

Invasive mechanical ventilation (IMV) is routinely prescribed to alleviate respiratory distress by decreasing the work of breathing to allow for patient recovery. These patients, requiring ventilatory intervention for respiratory diseases, are often concurrently treated with inhalation therapy [1,2]. With advances in technology and research focussing on gaining a better understanding of aerosol therapy during mechanical ventilation, this combined therapy is utilised worldwide [3]. Delivery of lung-targeting medications is facilitated using different types of aerosol-generating devices. The clinical utility of aerosol drug delivery during mechanical ventilation is influenced by the efficiency of these drug delivery devices and the ability to optimise the potential aerosol available for inhalation with respect to the artificial airways, humidification type and flow patterns within the ventilatory circuit [4,5].

Depending on the device selected for treatment, there are limitations associated with their use during mechanical ventilation [6]. Jet nebulisers (JNs) require a separate compressed gas source to operate, typically 6–8 L/min. This extra flow in the ventilatory circuit can change ventilatory parameters such as tidal volume [7]. Previous studies also showed that positive expiratory end pressure (PEEP) decreased within the pressurised ventilatory circuit when the JN was disconnected from the circuit during drug refill [8]. Additionally, large residual volumes of concentrated drugs can remain in the medication cup of a JN after nebulisation [9]. Increasing the fill volume will allow for greater drug delivery but will result in longer nebulisation times [10]. The practicalities surrounding the use of pressurised metered-dose inhalers (pMDIs) during mechanical ventilation also pose issues for caregivers. In clinical practice, the efficiency of drug delivery depends on the optimum technique of administration [11]. pMDIs are also limited to the low number of formulation types that are formulated in pMDIs. For example, antibiotics and mucolytics require nebulisers for drug delivery [12]. Vibrating mesh nebulisers (VMNs) are considered closed systems and, by design, can remain in the ventilatory circuit for up to 28 days; therefore, they do not require the circuit to be broken for drug refill. These nebulisers also have shorter treatment times and do not add any extra airflow to the closed circuit during operation.

Ari et al. (2015) [13] highlighted that the delivery efficiency of a pMDI, expressed as a percentage of the nominal dose, was greater than a JN during mechanical ventilation even though the JN delivered a greater inhaled drug mass. Another study by the same group showed that drug delivery using a VMN was higher than a JN at two different positions within the circuit, on the inspiratory limb and before the humidifier [14]. Further studies have shown that the type of aerosol device, device placement within the circuit and the presence of humidification all influenced drug delivery efficiency [15]. In the aforementioned study, a VMN delivered a higher percentage nominal drug dose in comparison to a JN and a pMDI when placed between the wye and endotracheal tube (ETT) and 15 cm from the wye in a humidified circuit. Additionally, the pMDI delivered a greater drug dose than a JN when placed 15 cm from the ventilator. Hatley and colleagues demonstrated that drug delivery using pMDIs can vary greatly depending on the time delay before actuation, which could lead to sedimentation of the drug within the device [16]. This was also noted in a recent study looking at actuation delay in albuterol hydrofluoroalkane (HFA) with a pMDI [17]. 

With the emergence of highly transmissible infectious diseases such as COVID-19, there is a requirement for infection control policies to be implemented for the safe administration of aerosolised drugs. The potential transmission of these viruses from patients in care environments can be detrimental. Published guidelines for the clinical management of COVID-19 patients have stated that there is insufficient evidence available to determine if the use of nebulisers contributes to the transmission of this disease [18]. pMDIs have been suggested as the device of choice for use with COVID-19 patients; however, there is no evidence to suggest that there is less risk associated with these devices [19]. Expert guidelines outline that treatment with medical aerosols is a low-risk procedure and does not increase the risk of disease transmission unless contamination has occurred from patients or caregivers [19]. 

The guidance around the humidification strategy during the COVID-19 pandemic has also evolved. Initially, a passive, filtered heat moisture exchanger (HME(F)) was suggested suitable for use on the basis that sufficient humidity would be delivered to the patient whilst, at the same time, minimising the risk of patient-derived bioaerosol emissions from the system. Practice rapidly evolved, however, and reverted to a mix of passive humidification and active, heated water column humidification. This was on the basis of the progression of the clinical course and other considerations [20]. With respect to aerosol delivery, both approaches require alternative aerosol generator placements and configurations, and the effect on the delivered dose at the end of the ETT has, thus, not been described during lung-protective ventilation.

Several recent studies by our group and others have quantified the risk of bioaerosol emissions from a simulated patient in the air during drug refill with a JN and have also found visual evidence, using the Schlieren optical method, of the release of patient-derived bioaerosol and fugitive drug aerosols from a IMV circuit during drug delivery with a pMDI and JN [8,21]. In these circumstances, health care workers should exercise caution by wearing appropriate personal protective equipment to reduce the risk of transmission. It should be noted that no such emissions were measured or observed during drug delivery or refill of a VMN [8,21]. O’Toole et al. [22] demonstrated that the placement of a filter on the exhalation port during mechanical ventilation mitigated the risk of potentially infectious fugitive emissions escaping from the circuit during nebuliser therapy.

Here, considering the multitude of recommendations around mechanical ventilation and aerosol drug delivery during the COVID-19 pandemic and the obvious importance of achieving optimal drug dosing for the benefit of the patient, for the first time, this study characterises the aerosol drug delivery efficiency of VMNs, JNs and pMDIs during standard (STD) and lung-protective low tidal volume (LTV) ventilation strategies in a simulated adult patient with both active and passive humidification. We also examine the effect of pMDI actuation timing on drug delivery. The residual drug mass within the devices and the pMDI adapters after drug delivery were also assessed.

## 2. Materials and Methods

### 2.1. Drug Delivery Devices

Experiments were conducted using the following devices: a VMN (Aerogen Solo, Aerogen Ltd., Galway Ireland), a JN (Airlife Misty Max 10, Vyaire Medical, Mettawa, IL, USA), and a pMDI (Salamol CFC-free Inhaler, TEVA Pharmaceuticals, Waterford, Ireland). Volumetric median diameter (VMD) as a measure of aerosol droplet size produced by the VMN and JN were measured using laser diffraction (Spraytec, Malvern Instruments, Malvern,, UK), as previously described [23].

### 2.2. Drug Delivery Determination

**Figure 1:**
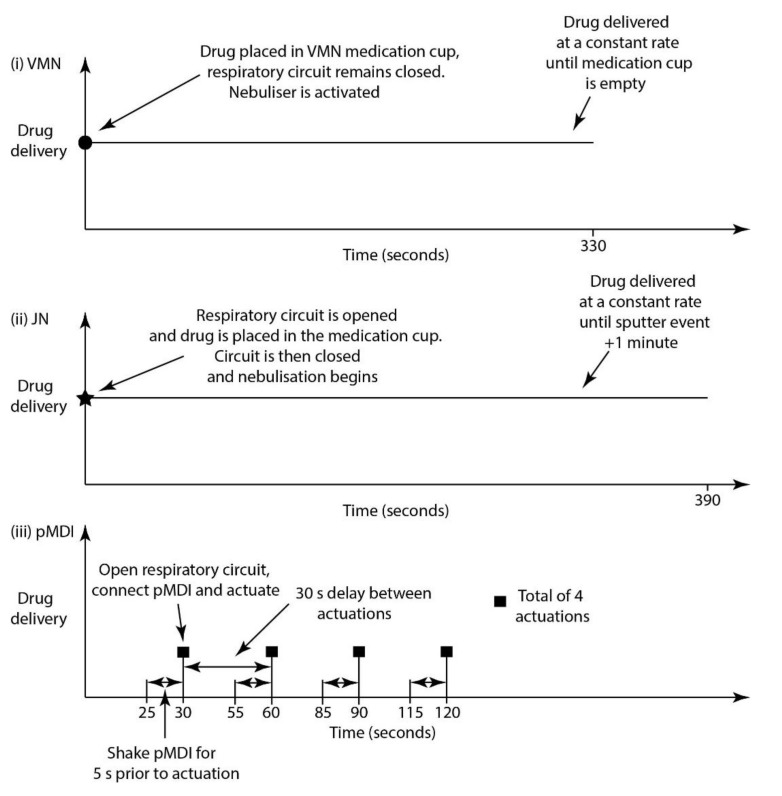
Stages in aerosol drug delivery to a mechanically ventilated patient via (**i**) VMN, (**ii**) compressed air-driven JN and (**iii**) pMDI.

For all testing with the VMN and JN, a 2500 µg dose of albuterol sulphate was administered at a concentration of 1 mg/mL, which represents the drug strength commercially available in Europe. For the pMDI, the device was primed before testing by discharging 4 doses from the device. Standard clinical practise recommends that each actuation of the device be delivered at the start of the inspiratory phase. A total of 4 actuations (400 µL), with a 5 s shake before each actuation and a 30 s delay between actuations, are to be delivered [24]. The different stages in the aerosol drug delivery process for the three devices are presented in Figure 1. After each test run, the mass of drug captured on the inhalation and exhalation filters (Respirgard 303EU, Vyaire, Basingstoke, UK) or spacer/connector was determined using UV spectrophotometry WPA lightwave II, Biochrom Ltd., Cambridge, UK) at 276 nm and interpolation on a standard curve of albuterol sulphate. All filters or spacer/connectors were washed in 10 mL of deionised water. The standard curve range was 3.125 to 100 mg/mL, with a recorded R^2^ of 0.9998. Drug recovery using this method and across both filter and spacer/connector was shown to be 100 ± 5%.

### 2.3. Aerosol Dose Efficiency during Simulated Invasive Mechanical Ventilation 

**Figure 2:**
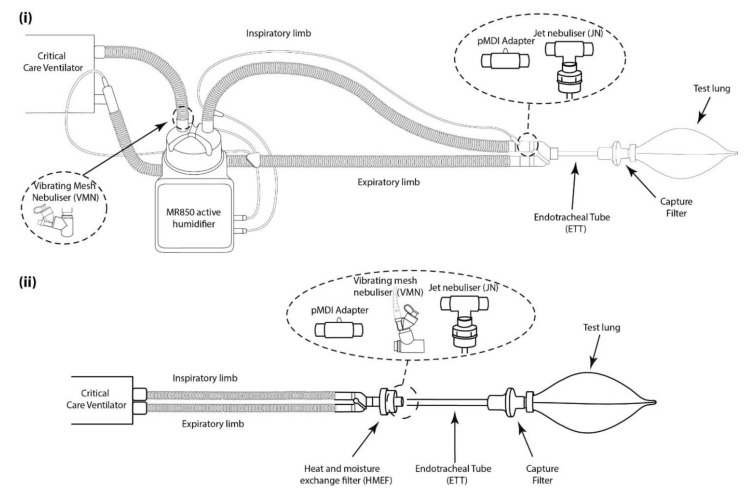
Illustration of simulated mechanical ventilation setup with (**i**) active humidification and (**ii**) passive humidification.

An illustration of the experimental setup is presented in Figure 2. A critical care mechanical ventilator (Servo-i, Maquet, Rastatt, Germany) incorporating a dual limb circuit (RT200, Fisher & Paykel (F&P), Auckland, New Zealand) was used with either active humidification (MR850 humidifier, Fisher & Paykel, Auckland, New Zealand) or passive humidification (heat moisture exchange filter (HMEF), Intersurgical, Wokingham, UK) placed at the patient side of the wye. A capture filter (Respirgard 303, Vyaire, Chicago, IL, USA) was attached between the endotracheal tube (ETT) and a test lung (SmartLung 2000, IMT Analytics, Buchs, Switzerland). A simulated standard adult ventilation pattern (Vt 500 mL, 15 BPM, and I:E ratio 1:1) [25] and an LTV ventilation pattern (Vt 400 mL, 20 BPM, I:E ratio 1:2) were assessed using all three devices. The VMN was placed at the dry side of the humidifier or between the HMEF and the ETT (8.0 mm, Flexicare, Mountain Ash, UK). The compressed air-driven (at 8 LPM) JN was placed on the inspiratory limb when using the humidifier or between the HMEF and the ETT. Three different pMDI adapters were assessed: (1) MiniSpacer (Hamilton Medical, Switzerland), (2) CombiHaler (OptimHal-ProtecSom, Valognes, France), and (3) Wye RT200 (Fisher & Paykel, Auckland, New Zealand). Each adapter was placed on the inspiratory limb [21]. For the wye adapter, the dual limb circuit used incorporated the facilitation of pMDI actuation in a port on the wye.

### 2.4. Residual Drug Determination

The residual drug mass in the VMN and JN was calculated gravimetrically. The nebulisers were weighed using an analytical balance (Ohaus Balance PR Series, Nanikon, Switzerland) pre- and post- nebulisation to determine the residual drug quantity remaining in the nebuliser cup after nebulisation. Any excess drug was removed from the outside of the devices before weighing, using lint-free absorbent paper, in order to ensure that only the drug remaining in the medication cup was accounted for. For the VMN, this was determined after nebulisation cessation; for the JN, it was determined after splutter occurrence plus 1 min. Concentration determination of the drug remaining in the JN was completed by UV spectrophotometry. The residual drug mass was also measured for the pMDI adapters using UV spectrophotometry at 276 nm. The residual drug remaining was expressed as a percentage of the nominal dose.

### 2.5. Ventilator Circuit Pressure Determination 

A pressure sensor (Citrex H5, IMT Analytics, Buchs, Switzerland) was placed between the wye and the ETT. The pressure in the circuit was monitored over a 24 s time interval to assess the change in circuit pressure during drug refill of the VMN and JN. Any potential pressure changes in the circuit during pMDI actuation and HME filter change were also assessed. Normal circuit pressure was monitored during the first eight seconds before and during the remaining time period after nebuliser drug refill, pMDI actuation or HME change.

### 2.6. Data Analysis

Statistical analysis was completed using GraphPad Prism. Unpaired *t*-test analysis was completed between VMN and JN devices, and one-way ANOVA (analysis of variance) tests were conducted between pMDI spacers/adapters to determine statistical significance between the test scenarios. Statistical differences were considered at *p* ≤ 0.05. Results are presented as mean ± standard deviation of drug delivered in µg. All testing was completed with five independent tests (*n* = 5).

## 3. Results

### 3.1. Droplet Size Determination

Aerosol droplet size characterisation of the delivery devices under test are presented in Table 1. Results are within the range of previously reported data.

### 3.2. Effect of pMDI Actuation Timing during the Respiratory Cycle on Drug Delivery

**Figure 3:**
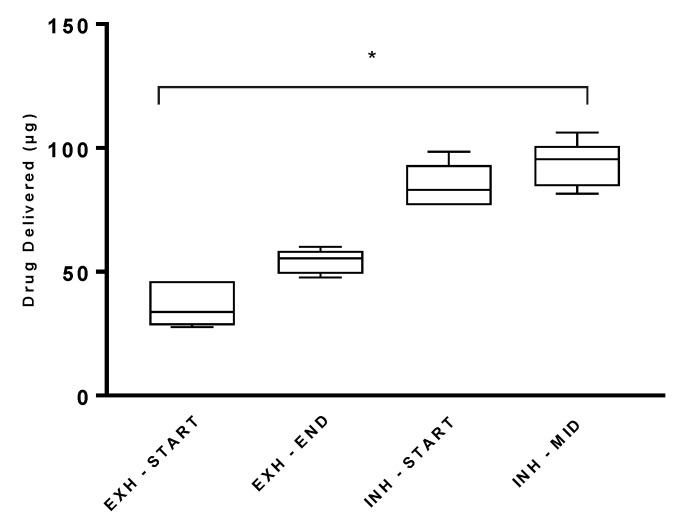
Drug delivery from pMDI actuation at different stages of the respiratory cycle (INH = inhalation; EXH = exhalation) during simulated mechanical ventilation with active humidification. Testing was completed with the pMDI and the MiniSpacer. Results are presented as mean ± SD µg drug delivered. * Denotes statistical significance at *p* < 0.0001.

Figure 3 presents the effect of actuating the pMDI at different stages of the inhalation/exhalation cycle on drug delivery during simulated mechanical ventilation. The highest drug delivery was achieved when the pMDI was actuated at the midway point of inhalation, 93.23 ± 9.27 µg. The clinical recommendation is to actuate the pMDI at the start of inhalation; this resulted in 84.62 ± 8.97 µg of drug being delivered. There was no statistically significant difference (*p* = 0.1733) in the drugs delivered at these two stages of inhalation. pMDI actuation at the end of exhalation resulted in a significantly lower delivered dose, 54.15 ± 4.91 µg (*p* = 0.0002 for start of inhalation; *p* = < 0.0001 for mid-inhalation). The lowest drug delivery was obtained when the pMDI was actuated at the start of exhalation, 36.62 ± 9.00 µg, and had a significant difference when compared with actuation at different points in the respiratory cycle (*p* < 0.0001 for both start and mid-inhalation; *p* = 0.0050 for end exhalation). 

### 3.3. Aerosol Drug Delivery during Simulated Mechanical Ventilation

#### 3.3.1. Standard Ventilation

**Figure 4:**
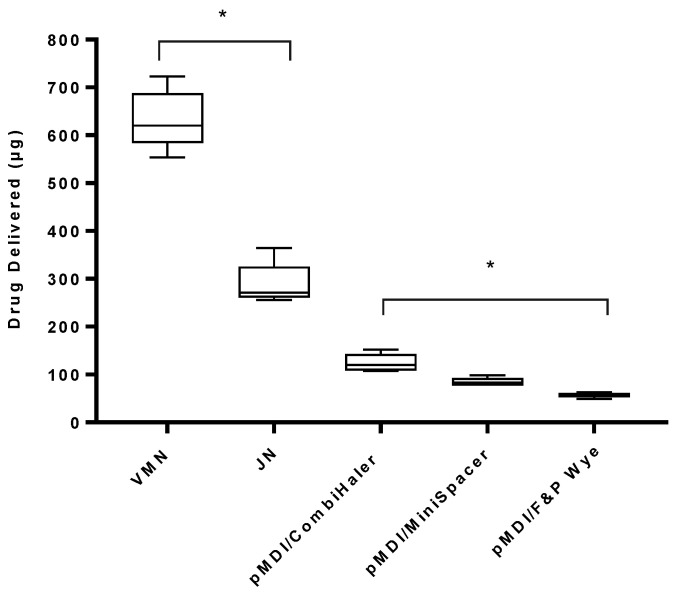
Aerosol drug delivery using standard adult settings during simulated mechanical ventilation with active humidification. Three different devices, VMN, JN and pMDI, using three different spacer/adapter types, were tested. pMDI actuation was at the start of inhalation. Results are presented as mean ± SD µg drug delivered. * Denotes statistical significance at *p* < 0.0001.

Table 2 presents the percentage of drug delivered during the different test scenarios. Figure 4 presents the mass of drug delivered to the end of the ETT using standard adult settings during invasive mechanical ventilation with active humidification. For the VMN, a result of 632.95 ± 61.95 µg was measured, whereas the JN delivered a significantly lower (*p* < 0.0001) dose of 288.60 ± 43.90 µg. For the pMDI, the quantity of drug delivered depended upon the type of in-line adapter used in the respiratory circuit. The highest dose delivered was measured using the CombiHaler spacer, 124.62 ± 18.68 µg, followed by the MiniSpacer, 84.62 ± 8.97 µg, and then the F&P wye, 56.92 ± 5.22 µg. Statistical significance between the adapters was determined at *p* < 0.0001. Aerosol delivery was significantly greater (*p* < 0.0001) using the VMN when compared with the highest yielding drug delivery using the CombiHaler spacer with the pMDI. This was also comparable with the JN (*p* < 0.0001).

**Figure 5:**
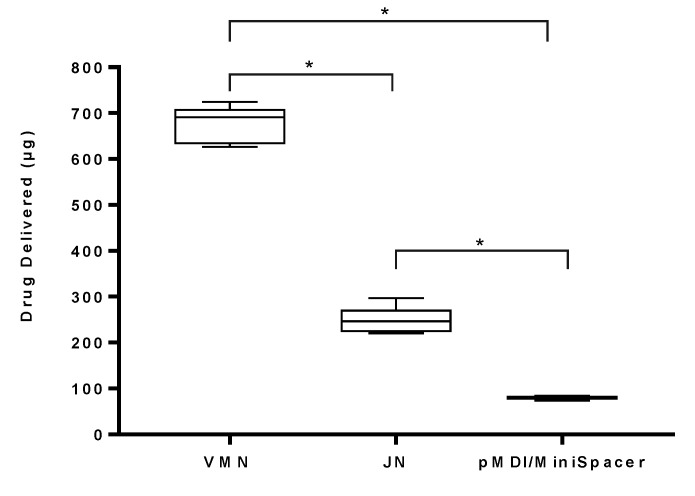
Aerosol drug delivery using standard adult settings during simulated mechanical ventilation with passive humidification (HMEF). Three different devices, VMN, JN and pMDI, using a MiniSpacer, were tested. pMDI actuation was at the start of inhalation. Results are presented as mean ± SD µg drug delivered. * Denotes statistical significance at *p* < 0.0001.

The mean ± standard deviation values of the mass of drug delivered during simulated mechanical ventilation using passive humidification are outlined in Figure 5. There was significantly greater (*p* < 0.0001) drug delivered using the VMN (674.77 ± 41.19 µg) in comparison to the JN (247.08 ± 30.24 µg) and the pMDI (80.00 ± 4.49 µg). A significant difference was also observed between the JN and pMDI (*p* < 0.0001). Overall, there was no statistically significant difference between passive and active humidification across all devices (*p* = 0.2440 for VMN, 0.1196 for JN and 0.3350 for pMDI).

#### 3.3.2. Lung-Protective, Low Tidal Volume (LTV) Ventilation 

**Figure 6:**
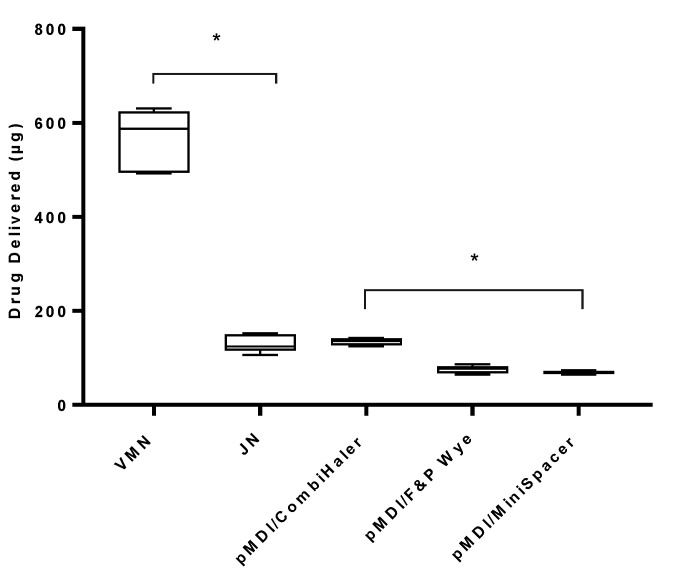
Aerosol drug delivery using LTV ventilation during simulated mechanical ventilation with active humidification. Three different devices, VMN, JN, and pMDI, using three different spacer/adapter types, were tested. pMDI actuation was at the start of inhalation. Results are presented as mean ± SD µg drug delivered. * Denotes statistical significance at *p* < 0.0001.

Table 2 presents the percentage of drug delivered during the different test scenarios. Figure 6 illustrates the mass of drug delivered during mechanical ventilation employing the lung-protective, LTV ventilation strategy with active humidification. In this test scenario, the VMN delivered the largest drug dose at 565.23 ± 66.36 µg in comparison to the JN at 131.08 ± 18.92 µg (*p* < 0.0001). For this series of testing, the adapter used influenced the quantity of drug delivered, with the pMDI/CombiHaler combination delivering the greatest drug dose, 134.46 ± 33.62 µg. Using the pMDI with the F&P wye resulted in 75.38 ± 8.14 µg of drug delivered and 68.92 ± 3.67 µg with the MiniSpacer. Statistical significance between the adapters was determined at *p* < 0.0001. With regards to the use of an LTV ventilation strategy in comparison to the standard ventilation settings, there was no significant difference when using the VMN (*p* = 0.1340) or the pMDI with the CombiHaler (*p* = 0.3067). However, using the JN resulted in a significantly lower delivered dose (*p* < 0.0001). For the pMDI with the F&P wye, the LTV ventilation yielded a significantly higher delivered drug dose (*p* = 0.0028) than standard ventilation settings. In contrast, the pMDI with the MiniSpacer delivered a significantly lower drug dose (*p* = 0.0068).

### 3.4. Percentage Residual Drug Remaining in the VMN and JN Devices

Table 3 presents the residual drug remaining in the medication cups of the VMN and JN and the pMDI adapters after drug delivery, expressed as a percentage of the nominal dose. For the VMN, using standard and LTV ventilation with active and passive humidification, there was <0.1% residual drug remaining in the device. However, using the JN, there was a considerable quantity of drug remaining post-nebulisation: 56.15–63.46% residual drug. The average drug concentration of the remaining residual drug was 1.2 mg/mL for an initial drug concentration of 1 mg/mL. This indicates that the drug had concentrated during nebulisation. For both the VMN and JN, there was no significant difference between the humidification types (*p* ≥ 0.05 for all test combinations). Results indicate that large quantities of drug remained in the spacers/adapters after actuation. The use of the CombiHaler and F&P wye with the pMDI resulted in the largest percentage residual drug, with no significant difference across both ventilation strategies applied. The MiniSpacer had the least percentage of drug remaining. For this adapter, there was a significant difference calculated across humidification type and ventilation type.

### 3.5. Exhaled Drug Determination during Simulated Mechanical Ventilation

Table 4 presents the drug captured on the filter placed on the exhalation limb during simulated mechanical ventilation with active humidification. For standard and LTV ventilation for device comparison, there were significant differences in drug loss through exhalation between the VMN and JN (*p* = 0.0319 for STD; *p* = < 0.0001 for LTV). In comparing ventilation parameters, there was a significantly greater percentage of drug exhaled when using LTV with a JN (*p* = 0.0007). In contrast, this was the case for standard ventilation using a VMN (*p* = 0.0449). With regards to the pMDI, there was no significant difference between spacers/adapters used (*p* = 0.1703) of the exhaled drug using standard ventilation. Using LTV ventilation, there were significant differences between the spacers/adapters used (*p* = 0.0001).

### 3.6. Effect of Nebuliser Refill/pMDI Actuation Use/HME Change on Circuit Pressure

**Figure 7:**
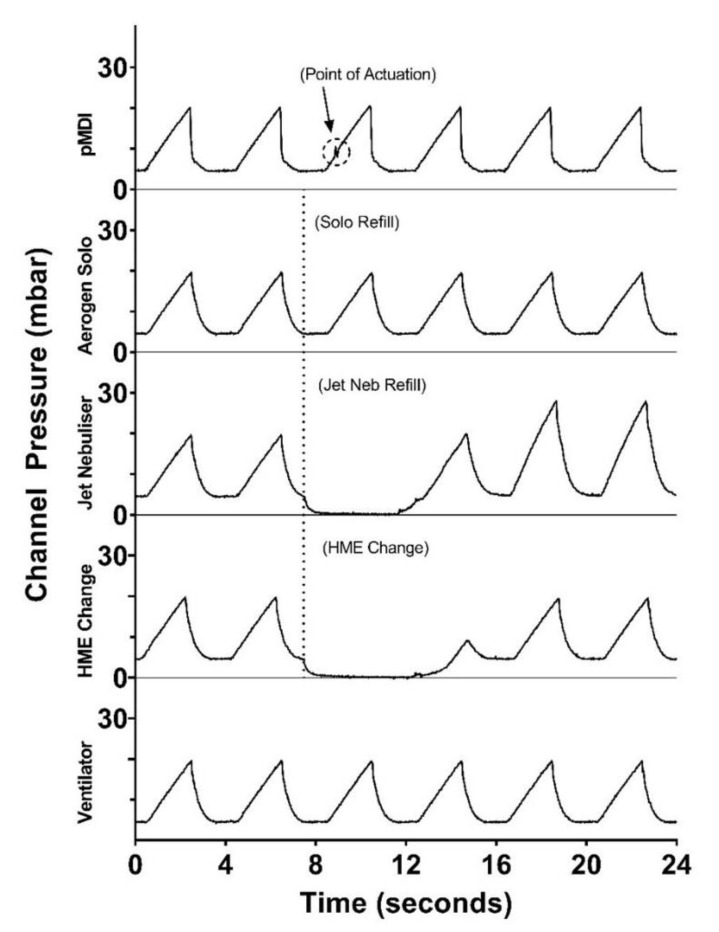
Ventilator circuit pressure change during nebuliser drug refill, pMDI actuation and HME change. Testing was completed using a pressure sensor. Results are presented as circuit pressure in mbar over time in seconds.

Figure 7 shows the change in respiratory circuit pressure during the drug refill of the VMN and JN and the actuation of the pMDI into the circuit. This testing also monitored the pressure change during the HME interchange in the circuit. Typically, HMEs would be changed in a clinical setting every 24–48 h [27]. For comparison, the pressure detected in the circuit with only the ventilator operating was approx. 4.4–20 mbar during the respiratory cycle. For the JN, there was a measured decrease in pressure during nebuliser drug refill, and it took 5 s for the pressure to revert to normal. There was also a decrease in the circuit pressure during the HME change, but, in this instance, it took 6.5 s for the pressure to return to normal. For the pMDI, there was a change in circuit pressure during actuation. However, this change was minimal and did not warrant any issue with respiratory circuit pressure. For the VMN, there was no change in circuit pressure during drug refill.

## 4. Discussion

Inhalation therapy using aerosol drug delivery devices is routinely used during mechanical ventilation in the treatment of respiratory diseases. This study examines the efficiency of aerosol drug delivery using VMNs, JNs and pMDIs to a simulated adult patient during standard adult and LTV ventilation with active and passive humidification. 

### 4.1. Effect of Device Type on Drug Delivery

This study shows that aerosol delivery to a simulated adult patient during mechanical ventilation is influenced by the type of aerosol drug delivery device selected, with the VMN delivering the highest drug dose across all testing conducted (see Figure 4, Figure 5 and Figure 6). These findings are in agreement with previous works by Ari and Fink (2021) [28], who reported a drug delivery of 23% from the VMN compared to just 7% for a JN during mechanical ventilation with active humidification. One study showed that the use of a VMN in the emergency department (ED) resulted in a reduction in hospital admissions, length of stay in the ED and albuterol drug dose when compared to a JN [29]. This current study recorded a larger residual percentage drug mass post-nebulisation with a JN (56–64%) in comparison to the VMN (<0.1%). The remaining residual drug in the JN was also concentrated when compared to the original drug concentration. Similar findings were also documented by Lengsfeld and Filas, 2008, [30], where JNs were measured to have 54–64% residual drug mass remaining post-nebulisation. In line with previous reports, the mass of drug delivered for both pMDI and JN was similar in many of the test conditions here [31]. These may go towards explaining the clinical observations wherein pMDIs were seen to be comparable or superior to “nebulisers” in ventilated patients [32]. This raises the important point that distinction needs to be made between the different types of nebulisers and more attention paid to the specific type reported in the literature. 

### 4.2. Effect of Ventilator Parameters and Humidification Type on Drug Delivery

Lung-protective LTV ventilation strategies are recommended for use in the treatment of severe respiratory disease in COVID-19 patients [33,34]. This current study compared standard and LTV ventilation parameters and their effects, if any, on drug delivery. The LTV ventilation parameters with active humidification did not affect the delivered dose using a VMN (632.92 ± 61.95 µg for STD vs. 565.23 ± 66.36 µg for LTV) or a pMDI/CombiHaler combination (124.62 ± 18.68 µg for STD vs. 134.46 ± 7.58 µg for LTV) with no significant difference detected. For a JN, there was a statistically significant difference in the delivered dose, with the use of LTV ventilation resulting in a lower delivered dose (288.60 ± 43.90 µg for STD vs. 131.08 ± 18.92 µg for LTV). With regards to humidification type, there was no statistical difference between the use of active or passive humidification on drug delivery across all device types with standard adult ventilation. Ari and colleagues [35] found that using a nonfiltered HME led to a greater drug delivery when compared to a filtered HME. There are HMEs that are designed to bypass the filter during aerosol delivery. Previous studies have investigated the use of these HME types and have shown that aerosol delivery is reduced in comparison to no HME present in the circuit [36]. 

### 4.3. Effect of pMDI Adapter Type on Drug Delivery

Spacers and adapters have been designed to facilitate increased drug delivery from pMDIs to mechanically ventilated patients as these inhalers, on their own, are not equipped for this functionality. The effect of a spacer/adapter on drug delivery was assessed using three different types of spacers/adapters. The CombiHaler spacer is designed as an inhalation chamber and can be connected to the circuit for ease of use with a pMDI [37]. Use of this spacer resulted in the largest quantity of drug delivered for both standard (124.62 ± 18.68 µg) and LTV (134.46 ± 7.58 µg) ventilation settings with active humidification. Consistent with previous studies, Boukhettala and colleagues [38] reported a three-fold increase in drug delivered using the CombiHaler with a pMDI in comparison to the MiniSpacer. A study by Eckles et al. (2020) [39] also reported a two- or three-fold increase in drug delivery using a pMDI with a spacer instead of a pMDI adapter. For standard ventilation settings with active humidification, the pMDI/MiniSpacer delivered a larger dose (84.62 ± 8.97 µg) in comparison to the pMDI/F&P wye (56.92 ± 5.22 µg). For LTV ventilation, the opposite was observed, with the pMDI/F&P wye delivering a larger dose (75.38 ± 8.14 µg) in comparison to the pMDI/MiniSpacer (68.92 ± 3.67 µg). The greater quantity of drug delivered using the CombiHaler with the pMDI could be attributed to its larger size. This would allow for the dual effect of (a) reducing ballistic fraction losses as the high-velocity pMDI plume (30–60 m/s) [40,41] has a greater chance of entrainment in the gas flow and (b) the aerosol being held within the chamber and subsequently delivering a bolus of drug during the inspiratory phase. However, this may also be the reason why such a large residual drug mass remained in the spacer post-dosing. Here, the highest percentage residual drug mass was measured with the F&P wye with a pMDI port. As the pMDI port is located directly on the wye, the larger residual drug mass is most likely a combination of the drug which may have remained during actuation (the ballistic fraction) and the drug deposited during exhalation.

### 4.4. Effect of pMDI Actuation Timing on Drug Delivery

The method employed for pMDI actuation into a ventilatory circuit is important for optimum drug delivery. Here, we investigated the actuation timing at different stages of the respiratory cycle. This study demonstrates that pMDI actuation timing can lead to variability in potential drug delivery. The largest delivered dose was achieved with actuation midway through the inhalation phase. However, there was no statistically significant difference between this point and the clinical recommendation of actuation at the start of inhalation. Actuation during inhalation resulted in a 1.5–2.5-fold increase in the quantity of drug delivered in comparison to actuation during the exhalation phase. In a previous study by Diot et al. [31], it was reported that there was a reduction of 35% in inhaled drug mass when it was not synchronised with inhalation. Of note, this variability in pMDI performance has been well described for decades and yet remains unresolved through either training or design [42,43].

### 4.5. Effect of Open Circuit

This study investigated the pressure change in the ventilatory circuit during the drug refill of the VMN and JN and the actuation of the pMDI into the circuit. Using a JN, the respiratory circuit must be broken to allow for drug placement in the medication cup of the nebuliser prior to delivery to the patient. In contrast, a VMN can remain in the circuit for 28 days and does not require the circuit to be broken for drug refill. To facilitate drug delivery with a pMDI, a port on the spacer or adapter must be opened to allow for actuation. There was a decrease in the circuit pressure recorded during the drug refill of the JN, but no change was measured during the refill of the VMN. This is in agreement with a previous study that showed a decrease in PEEP, from 4.5 to 0 cm H_2_O, during the drug refill of a JN [8]. Actuation of the pMDI did cause a drop in pressure, indicating that the circuit had been broken open and potentially pathogen-laden air had escaped. 

In clinical practice, the recommendation to remove HMEs either prior to aerosol therapy or at the end of its use life results in disconnection from the ventilator circuit, which interferes with the respiratory circuit pressures [44]. In this current study, disconnection of the circuit for HME removal and insertion resulted in a decrease in pressure to 0 mbar and required 6.5 s to return to normal circuit pressures. The risk of infection to the patient and the healthcare worker can also increase with patient disconnection from the ventilator to facilitate aerosol drug delivery or HME exchange. In confirmation of this theory, a visual investigation was undertaken to demonstrate the release of potentially infectious patient-derived bioaerosol or fugitive drug emissions during aerosol drug delivery using different devices during mechanical ventilation [21]. In this study, it was observed that a combination of ventilator air, patient-derived bioaerosol and fugitive drug emissions were released during aerosol therapy using a pMDI and JN but not with a VMN. Joyce et al. [8] also demonstrated the release of patient-derived bioaerosol with an aerosol particle sizer detecting a median particle count of 710 per cm^3^ above ambient during the drug refill of a JN in comparison to 0 particles per cm^3^ for a VMN during mechanical ventilation. O’Toole et al. [22] examined the effect of filters on the exhalation port of the dual limb circuit in preventing the release of fugitive drug emissions from a simulated mechanically ventilated patient. The authors found that placing a filter on the exhalation port mitigated the risk of fugitive drug emissions escaping from the closed circuit.

## 5. Conclusions

In this study, we have shown that device type and ventilator parameters influence drug delivery during simulated adult mechanical ventilation. Humidification type had no influence on drug delivery using standard breath/ventilator settings. This study highlights the variability in drug delivery using a pMDI and that drug delivery technique and spacer/adapter choice are critical factors to be considered when using this device as a treatment option. These results would suggest that contrary to some guidance promoting their use, pMDIs are, indeed, not fit for purpose with respect to optimal drug delivery to the patient’s lungs under either standard or the now widely adopted lung-protective LTV ventilation strategy used in the COVID-19 patient. Considering their low dose delivery, variability in performance and the fact that pMDIs are not general-purpose, i.e., they have a specific formulation packaged inside and are not suitable for use with other formulations without significant reformulation, pMDIs do not represent a viable option for the rapid pandemic-driven development of novel or repurposed therapeutics. This is further supported by the literature, as discussed above, when one considers the requirement to break the ventilator circuit for pMDI drug administration. Conversely, this study supports the various clinical guidance documents that call for closed-circuit aerosol delivery systems in an effort to minimise the risk of escape of patient-derived bioaerosols [45,46,47]. This current research will increase the understanding of aerosol drug delivery across different drug delivery devices during standard and LTV ventilation utilising active and passive humidification. 

Further, the results reported here may go towards informing appropriate dosing strategies and device choice for the development, investigation, and therapeutic administration of formulations in mechanically ventilated patients. It should be noted that the nebulisers used in this study were current, commercially available general-purpose devices. Further optimisation of lung dose may be possible through the use of more sophisticated devices such as those that make use of breath actuation or tailored/smaller droplet sizes [48,49,50,51]. This may be a critical consideration in the administration of novel, high-value therapeutics, such as advanced therapeutic medicinal products (ATMPs) or those with associated adverse side effects when suboptimal doses are delivered [52,53,54,55,56].

## Figures and Tables

**Table 1:** Droplet size characterisation of aerosol delivery devices.

Device Type	VMD (µm)	Flow Rate (mL/min)	FPF <5 µm (%)	FPF <3 µm (%)	FPF <2 µm (%)
VMN	4.82 ± 0.06	0.56 ± 0.00	48.03 ± 0.69	27.60 ± 0.64	15.33 ± 0.64
JN	3.42 ± 0.02	0.27 ± 0.00	72.20 ± 0.27	42.53 ± 0.23	22.89 ± 0.03
pMDI	1.61 ± 0.18 ^a^	N/A	94.70 ± 0.34 ^a^	N/A	N/A

^a^ Reported data [26].

**Table 2:** Mean ± standard deviation drug delivered across three devices with differing ventilation strategies, humidification types and drug concentrations.

Device Type	Ventilation Type	Circuit Position	Humidification Type	Drug Delivery (µg) Mean ± SD	Drug Delivery (%) Mean ± SD
VMN	STD	Dry side	ACTIVE	632.92 ± 61.95	25.32 ± 2.48
		Between HME and ETT	PASSIVE	674.77 ± 41.19	26.99 ± 1.99
	LTV	Dry side	ACTIVE	565.23 ± 66.36	22.61 ± 2.65
JN	STD	Inspiratory limb	ACTIVE	288.60 ± 43.90	11.54 ± 1.76
		Between HME and ETT	PASSIVE	247.08 ± 30.24	9.88 ± 1.21
	LTV	Inspiratory limb	ACTIVE	131.08 ± 18.92	5.24 ± 0.76
pMDI/CombiHaler	STD	Inspiratory limb	ACTIVE	124.62 ± 18.68	31.15 ± 4.67
pMDI/MiniSpacer				84.62 ± 8.97	21.15 ± 2.24
pMDI/F&P Wye		At the wye		56.92 ± 5.22	14.23 ± 1.30
pMDI/MiniSpacer		Between HME and ETT	PASSIVE	80.00 ± 4.49	20.00 ± 1.12
pMDI/CombiHaler	LTV	Inspiratory limb	ACTIVE	134.46 ± 7.58	33.62 ± 1.90
pMDI/MiniSpacer				68.92 ± 3.67	17.23 ± 0.92
pMDI/F&P Wye				75.38 ± 8.14	18.85 ± 2.04

**Table 3:** Residual drug post nebulisation within the VMN and JN devices and the pMDI adapters during active and passive humidification (STD = standard). Results are presented as mean ± SD % residual drug.

Device Type	Humidification Type	Ventilation Type	Mean ± SD (% Residual Drug)	*p*-Value
VMN	ACTIVE	STD	0.05 ± 0.08	0.5447
	PASSIVE		0.02 ± 0.03	
	ACTIVE	LTV	0.01 ± 0.03	0.3589
JN	ACTIVE	STD	57.12 ± 3.83	0.0741
	PASSIVE		63.46 ± 5.75	
	ACTIVE	LTV	56.15 ± 12.20	0.8695
CombiHaler	ACTIVE	STD	56.46 ± 9.38	0.1333
		LTV	65.38 ± 7.40	
F&P Wye	ACTIVE	STD	64.46 ± 6.08	0.6585
		LTV	63.00 ± 3.70	
MiniSpacer	ACTIVE	STD	29.38 ± 2.35	<0.0001
	PASSIVE		18.46 ± 1.28	
	ACTIVE	LTV	17.23 ± 5.93	0.0028

**Table 4:** Mean ± standard deviation of drug (µg) captured on expiratory filter across three devices with differing ventilation strategies.

Device Type	Ventilation Type	Expiratory Filter Mean ± SD (µg Drug)	Expiratory Filter Mean ± SD (% Exhaled Drug)
VMN	STD	211.40 ± 35.90	8.46 ± 1.43
	LTV	162.80 ± 28.40	6.51 ± 1.14
JN	STD	273.20 ± 39.50	10.93 ± 1.58
	LTV	400.30 ± 36.30	16.01 ± 1.45
pMDI/CombiHaler	STD	33.54 ± 4.27	8.38 ± 1.07
	LTV	58.15 ± 6.10	14.54 ± 1.52
pMDI/MiniSpacer	STD	36.00 ± 7.43	9.00 ± 1.86
	LTV	37.54 ± 4.56	9.38 ± 1.14
pMDI/F&P Wye	STD	29.54 ± 7.49	7.38 ± 1.87
	LTV	48.92 ± 4.54	12.23 ± 1.13

## Data Availability

The data presented in this study are available on request from the corresponding author.
